# Heart Rate Variability and Pulse Rate Variability: Do Anatomical Location and Sampling Rate Matter?

**DOI:** 10.3390/s24072048

**Published:** 2024-03-23

**Authors:** Joel S. Burma, James K. Griffiths, Andrew P. Lapointe, Ibukunoluwa K. Oni, Ateyeh Soroush, Joseph Carere, Jonathan D. Smirl, Jeff F. Dunn

**Affiliations:** 1 Cerebrovascular Concussion Laboratory, Faculty of Kinesiology, University of Calgary, Calgary, AB T2N 1N4, Canada; james.griffiths1@ucalgary.ca (J.K.G.); joseph.carere@ucalgary.ca (J.C.); jonathan.smirl@ucalgary.ca (J.D.S.); 2 Libin Cardiovascular Institute of Alberta, University of Calgary, Calgary, AB T2N 1N4, Canada; 3 Alberta Children’s Hospital Research Institute, University of Calgary, Calgary, AB T2N 1N4, Canada; ibukunoluwa.oni1@ucalgary.ca (I.K.O.); ateyeh.soroush@ucalgary.ca (A.S.); dunnj@ucalgary.ca (J.F.D.); 4 Hotchkiss Brain Institute, University of Calgary, Calgary, AB T2N 1N4, Canada; 5 Integrated Concussion Research Program, University of Calgary, Calgary, AB T2N 1N4, Canada; 6 Sport Injury Prevention Research Centre, Faculty of Kinesiology, University of Calgary, Calgary, AB T2N 1N4, Canada; 7 Human Performance Laboratory, Faculty of Kinesiology, University of Calgary, Calgary, AB T2N 1N4, Canada; 8 Faculty of Biomedical Engineering, University of Calgary, Calgary, AB T2N 1N4, Canada; 9 Atlas Institute for Veterans and Families, Ottawa, ON K1Z 7K4, Canada; andrew.lapointe@ucalgary.ca; 10 Department of Clinical Neurosciences, Cumming School of Medicine, University of Calgary, Calgary, AB T2N 1N4, Canada

**Keywords:** heart rate variability, pulse rate variability, cerebral vasculature, peripheral vasculature, validity

## Abstract

Wearable technology and neuroimaging equipment using photoplethysmography (PPG) have become increasingly popularized in recent years. Several investigations deriving pulse rate variability (PRV) from PPG have demonstrated that a slight bias exists compared to concurrent heart rate variability (HRV) estimates. PPG devices commonly sample at ~20–100 Hz, where the minimum sampling frequency to derive valid PRV metrics is unknown. Further, due to different autonomic innervation, it is unknown if PRV metrics are harmonious between the cerebral and peripheral vasculature. Cardiac activity via electrocardiography (ECG) and PPG were obtained concurrently in 54 participants (29 females) in an upright orthostatic position. PPG data were collected at three anatomical locations: left third phalanx, middle cerebral artery, and posterior cerebral artery using a Finapres NOVA device and transcranial Doppler ultrasound. Data were sampled for five minutes at 1000 Hz and downsampled to frequencies ranging from 20 to 500 Hz. HRV (via ECG) and PRV (via PPG) were quantified and compared at 1000 Hz using Bland–Altman plots and coefficient of variation (CoV). A sampling frequency of ~100–200 Hz was required to produce PRV metrics with a bias of less than 2%, while a sampling rate of ~40–50 Hz elicited a bias smaller than 20%. At 1000 Hz, time- and frequency-domain PRV measures were slightly elevated compared to those derived from HRV (mean bias: ~1–8%). In conjunction with previous reports, PRV and HRV were not surrogate biomarkers due to the different nature of the collected waveforms. Nevertheless, PRV estimates displayed greater validity at a lower sampling rate compared to HRV estimates.

## 1. Introduction

Heart rate variability (HRV) describes the minute deviations in intervals between successive heartbeats [1,2]. A 3- to 12-lead electrocardiography (ECG) is typically used to quantify HRV, which can determine the electrical activity of the heart during atrial and ventricle depolarization and repolarization [1,3]. From this, one can identify the QRS complex, corresponding with ventricular contraction, where the R-R intervals are used to determine HRV metrics in time- and frequency-domains (e.g., standard deviation of successive N-N intervals [SDNN], the root mean square of successive differences between heartbeats [RMSSD], relative low frequency [LF], and relative high frequency [HF]) [1]. These metrics have demonstrated high clinical utility in their ability to discriminate pathophysiological differences between healthy and clinical populations (e.g., myocardial infarction, diabetes, renal failure) (reviewed in: [4]). With the constant development and expansion of technology, a plethora of commercial devices are being developed claiming they can capture cardiovascular activity during activities of daily life [5,6,7,8,9]. These claims are often stated without proper reporting of their validity. Consequently, it is imperative the validity and accuracy of different techniques are adequately appraised. An example of such a technology is the quantification of cardiac activity via photoplethysmography (PPG) [10].

Rather than measuring the electrical activity of the heart (i.e., ECG), PPG devices measure blood volumetric changes within the vascular bed of different anatomical locations [11]. This occurs downstream from the ventricular contraction, meaning a slight transit time delay exists between the “R-spike” that is captured with ECG and the “P-waveform peak” with PPG [11]. Moreover, an important physiological difference exists between waveforms. An ECG captures a short-lived QRS complex with a discrete peak lasting ~20 milliseconds that corresponds to the electrical activity of the heart during ventricular contraction [12]. Conversely, PPG captures changes in blood volumes within the microvascular bed, which consists of a rounded peak lasting ~30–40 milliseconds [11]. Previous work has begun to capture HRV from PPG; however, given the time delay and signal difference between ECG and PPG, it has been proposed that HRV and pulse-rate variability (PRV, i.e., HRV derived from pulsatile waveforms) may be correlated but PRV might not be a direct surrogate for HRV [13,14]. For example, a study of individuals with a fixed-rate pacemaker noted the presence of PRV, despite a lack of variation in HRV [15].

Further, as time delays exist due to vascular tone/vessel diameter, different anatomical locations may produce slightly different PRV metrics (e.g., cerebral artery, phalanges) [16,17]. This would be especially paramount for optical imaging techniques, such as continuous wave near-infrared spectroscopy (fNIRS) that can create >40 unique PPG channels during one recording session across a multitude of cortical regions [18,19]. If a transient timing difference is present between cerebral cortical locations, a PRV recording in the occipital region could produce slightly different estimates compared to the prefrontal cortices. Previous work denoted PRV differences in PPG recordings between the wrist and forearm on the same arm [17,20]. However, no investigations have compared the validity of PPG waveforms between the cerebral and peripheral vasculature with respect to the validity of PRV metrics, as the former has sympathetic and parasympathetic innervation, while the latter has only sympathetic innervation [21]. This is imperative as prior research has demonstrated the cerebral and peripheral vasculature respond differently to the same stimuli [22].

Given the physiological differences between what is measured with an ECG (PQRST complex) and PPG (pulsatile waveform), an investigation into the validity of PPG and its agreement with a “*gold-standard*” ECG when measuring HRV is fundamental [11]. This will help mitigate a besmirching of the literature with poorly designed PPG studies. Previous work has demonstrated sampling frequency is important when deriving HRV metrics from an ECG [23]. For example, Burma and colleagues [23] compared different ECG sampling rates against a *“gold-standard”* 1000 Hz ECG recording, where a sampling rate of 50 Hz was required to obtain a valid heart rate estimate with this device. However, HRV metrics displayed greater sensitivity to sampling rate, where a recording of 90 Hz was required for time- and frequency-domain estimates [23]. This was postulated to HRV being more sensitive to millisecond differences in R-spike detection, due to the short-lived nature of the QRS complex [12]. As previously mentioned, the waveform detected with PPG generally occurs over a slightly longer duration [11], and thus, it is unknown if sampling rate has an impact on PRV estimates. This is especially paramount, given the majority of smartwatches sample between 20 and 100 Hz [24,25,26].

HRV metrics have demonstrated high utility in research and clinical settings; however, while 24 h recordings can occur with ECG devices (i.e., Holter monitor), this approach requires participants to secure a monitoring device to their chest with three or more electrodes placed at various locations across the torso [1,4]. Conversely, a strength of PPG technology is that one would be able to record 24 h PPG recordings from a smartwatch with minimal intrusion into one’s daily life/activities [10]. Therefore, the purpose of this investigation was two-fold: (1) to compare the validity of PPG-derived PRV metrics at three anatomical locations (e.g., finger via finger PPG with blood pressure corrected to the level of the heart, middle cerebral artery velocity via transcranial Doppler ultrasound [TCD], and posterior cerebral artery velocity via TCD) against “*gold-standard*” ECG-derived HRV metrics from recordings at 1000 Hz; and (2) to delineate the minimum sampling rate required to obtain robust PRV metrics at the three aforementioned locations. Based on past literature, it was hypothesized that time- and frequency-domain PRV metrics would be greater from PRV compared to concurrent HRV recordings [17]. Further, due to the longer-lived nature of the P-waveform peak, compared to the R-spike, it was hypothesized that PRV would display greater validity at a lower sampling rate compared to HRV [11,12]. This will help determine the potential utility of smartwatches and wearable technology to robustly capture autonomic function during daily activities. 

## 2. Methods

### 2.1. Ethical Approval

The current study received ethical approval from the University of Calgary Conjoint Health Research Ethics Review Board (REB20-1662 and REB20-2112). All protocols completed were compliant with guidelines/recommendations put forth in the Declaration of Helsinki (revised version 2013), aside from item number 35 (i.e., registration of the study within a database) [27]. Prior to participation, study protocols were described with participants being able to ask any questions about the protocol and equipment utilized. Following this, participants provided written informed consent.

### 2.2. Study Design and Participants

This was a cross-sectional study, with all data being collected in one visit. This investigation followed similar guidelines as a recent report [23] but expanded to include pulsatile waveforms, as the use of this measure to quantify HRV/PRV with wearable technology has substantially grown over the last decade [10]. Participants free of cardiovascular, cardiovascular, respiratory, and neurological disease between the ages of 18–40 years were included in the current investigation. Individuals with cardiac arrhythmias or irregularities were excluded from the current analysis. Fifty-four individuals participated, consisting of 29 females and 25 males. Females were an average age of 25.9 ± 6.9 years with a body mass of 24.1 ± 2.8 kg/m^2^ and males were an average age of 24.3 ± 4.3 years with a body mass of 24.8 ± 1.6 kg/m^2^. Testing for female participants was completed during the early follicular phase (days 3–10) where hormones are known to be stable [28]. All testing was completed between the hours of 08:00–18:00, in a quiet laboratory setting [29]. Prior to testing, all individuals refrained from caffeine, nicotine, and alcohol for a minimum of 12 h [4,30], exercise for a minimum of two hours [29], and food for a minimum of one hour [31,32].

### 2.3. Instrumentation

A three-lead ECG (FE231, ADInstruments, Colorado Springs, CO, USA) was used with electrodes placed under the left collarbone, right collarbone, and laterally to the left of the navel. A Finapres NOVA device quantified beat-to-beat blood pressure pulsatile waveforms of the finger, which was corrected for heart height via a height correction unit (Finapres Medical Systems, Amsterdam, The Netherlands) [33,34]. Transcranial Doppler ultrasound (Doppler Box, DWL USA Inc., San Juan Capistrano, CA, USA) was used to insonate MCA and PCA, producing a cerebral blood velocity pulsatile waveform [35]. Carotid compressions and a basic visual tracking task were used to ensure the MCA and PCA were insonated, respectively [35]. TCD pulsatile waveforms were used in the current investigation due to this neuroimaging technique bolstering superior temporal resolution compared to others (e.g., fNIRS, functional magnetic resonance imaging [fMRI]) [35]. All data were collected and time-synchronized using a PowerLab device (PowerLab 16/35, ADInstruments, Colorado Springs, CO, USA), which samples at 1000 Hz [23].

### 2.4. Experimental Protocols

The current analysis is a subsection of a larger investigation seeking to better understand cerebrovascular and cardiovascular regulation. Participants were seated for ~30 min, before transitioning into an upright orthostatic position. The HRV/PRV recordings began ~60–120 s following this transition, allowing for all hemodynamic responses to normalize to the new orthostatic posture [36,37]. Following this, participants stood for 5 min while ECG and PPG data were collected [38].

### 2.5. Data Processing

**Figure 1:**
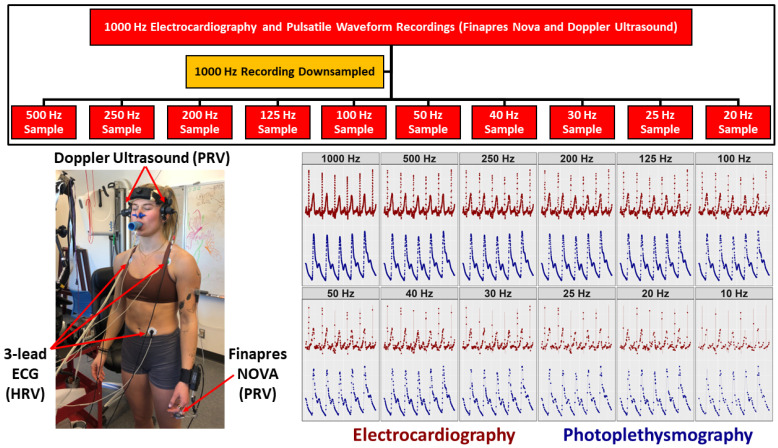
A flow chart depicting the study protocol of the 1000 Hz recordings being downsampled from their original 1000 Hz waveform to frequencies ranging from 20 to 500 Hz. A participant is shown wearing the equipment used to assess cardiac electrical activity via a 3-lead electrocardiogram (ECG) and pulsatile waveforms via transcranial Doppler ultrasound and Finapres NOVA. The 3-lead ECG captured heart rate variability (HRV) while the Doppler ultrasound and Finapres NOVA devices captured pulse rate variability (PRV). Further, the Doppler ultrasound was used to capture PRV in both the middle and posterior cerebral arteries. Finally, the bottom right displays representative data from one participant of their 1000 Hz ECG (dark red) and pulsatile waveforms (dark blue) at 1000 Hz. Data were then downsampled to various frequencies, illustrating the impact sampling rate has on the ability to robustly detect the “R-spike” and “P-spike”.

All 1000 Hz recordings were downsampled to sampling frequencies of 500, 250, 200, 125, 100, 50, 40, 30, 25, and 20 Hz (MATLAB 2022, v.9.9.0) (Figure 1) [39]. Python (*Python Software Foundation.* (*2023*)*. v.3.8.3*) was then used for peak identification and correction for the ECG and PPG waveforms on all sampled data [40]. Time- and frequency-domain analyses were computed to extract HRV and PRV metrics [1]. From these, the outcome metrics of interest were heart rate, SDNN, RMSSD, relative LF, relative HF, and LF/HF [1]. Band-averages were used to calculate the relative LF (0.04–0.15 Hz) and relative HF (0.15–0.40 Hz) frequency-domain metrics [1].

### 2.6. Sample Size Calculation

The data presented by Burma and colleagues [23] demonstrated large effect size differences in HRV metrics at 20 Hz compared to 1000 Hz, which required a sample of less than 10 to determine a within-individual difference. However, a more conservative approach was applied in the current investigation to ensure moderate effect size differences could be delineated between techniques and sampling frequencies. Therefore, an alpha of 0.05, power of 95%, Cohen’s *d* of 0.5 (small-to-moderate threshold), and two-tailed hypothesis testing were input into G*Power software (version 3.1.9.6), where a sample size of 54 participants was required.

### 2.7. Statistical Analysis

Statistical analyses were conducted with RStudio (v.2023.0.0) [41]. Prior to analysis, all outcome variables were log-transformed, as HRV data are known to be non-normally distributed [42]. Bland–Altman plots with 95% limits of agreement (LOA) were used to quantify absolute agreement [43]. Based on previous recommendations, differences between measures (i.e., *y*-axis) were turned into ratios to account for non-normal distribution and skewed data [44]. The *x*-axis on the Bland–Altman plots were displayed as the log-transformed mean due to the large variability from the lower downsampled frequencies making the absolute mean values difficult to compare within a figure. This was completed to compare the PRV estimates to the HRV estimates at 1000 Hz, as the latter is considered the “*Gold-Standard*”. This enabled an understanding of the potential bias that exists between HRV and PRV. Additionally, Bland–Altman plots with 95% LOA were used to compare the downsampled HRV/PRV data obtained within each waveform (i.e., ECG, BP, MCA, and PCA) to the 1000 Hz recording. This allowed for an understanding of the precise sampling frequency required to obtain valid HRV/PRV outcomes from each respective waveform. Moreover, coefficient of variation (CoV) and Cohen’s *d* effect sizes were computed to additionally compare these two aims. The CoV was calculated as the standard deviation of the two samples divided by the mean for each comparison [45]. From this, mean and 95% confidence intervals (95% CI) were computed. Thresholds of <5% (excellent), 5–10% (good), 10–20% (acceptable), and >20% (unacceptable) were used for CoV metrics [46,47]. Cohen’s *d* effect sizes determined the magnitude difference between comparisons with thresholds of <0.20 (negligible), 0.20–0.50 (small), 0.50–0.80 (moderate), and >0.80 (large) [48]. Effect sizes were utilized as these have been proposed to provide clinical relevance in physiological literature to a greater extent than a binary *p*-value [49,50,51]. Finally, linear regression was completed for each downsampled frequency relative to its own log-transformed 1000 Hz value. This identified the specific downsampled sampling rate where the 95% confidence intervals did not contain the 1000 Hz “reference-value”. Alpha was set *a priori* at 0.05. While numerous comparisons were performed, no alpha correction was completed as the purpose was to identify the lowest sampling rate required to produce valid estimates. Therefore, reducing the alpha would have led to incorrect physiological interpretations.

## 3. Results

### 3.1. Heart Rate Variability vs. Pulse Rate Variability at 1000 Hz

**Figure 2:**
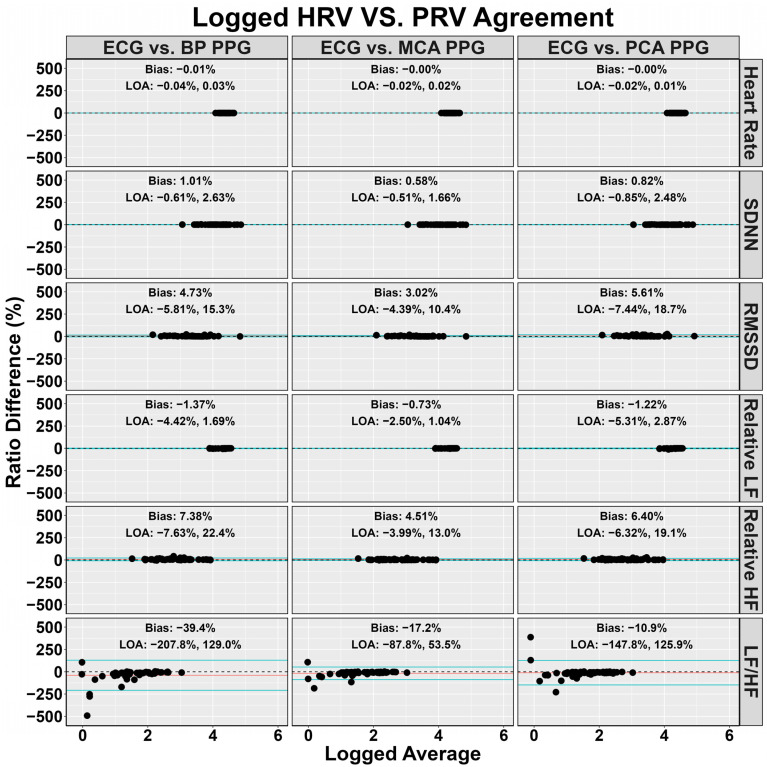
Log-transformed Bland–Altman plot with 95% limits of agreement depicting the validity of pulse rate variability compared to heart rate variability at 1000 Hz in 54 individuals (29 females and 25 males). The mean difference is shown with a red dashed line and the 95% limits of agreement are shown with the blue lines. Heart rate variability metrics were obtained from an electrocardiography (ECG) and pulse rate variability metrics from three devices using photoplethysmography (PPG) measuring blood pressure (BP) at the finger and velocity within the middle cerebral artery (MCA) and posterior cerebral artery (PCA). Differences were transformed to ratios due to non-normally distributed data. Standard deviation of N-N intervals (SDNN), root mean square of successive differences between heartbeats (RMSSD), low frequency (LF), and high frequency (HF).

**Figure 3:**
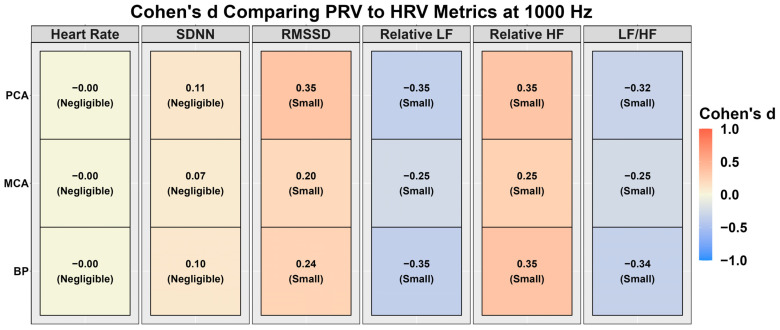
Cohen’s *d* effect size values comparing heart rate variability (HRV) metrics to pulse rate variability (PRV) at 1000 Hz in 54 individuals (29 females and 25 males). Heart rate variability metrics were obtained from an electrocardiography (ECG) and pulse rate variability metrics from three devices using photoplethysmography (PPG) measuring blood pressure (BP) at the finger and velocity within the middle cerebral artery (MCA) and posterior cerebral artery (PCA). Cohen’s *d* effect sizes determined the magnitude difference between comparisons with thresholds of <0.20 (negligible), 0.20–0.50 (small), 0.50–0.80 (moderate), and >0.80 (large). A negative value (blue) corresponds to a PRV metric that produced a higher value than the 1000 Hz recording, while a positive value (red) corresponds to a PRV metric that produced a lower value than the 1000 Hz recording. Standard deviation of N-N intervals (SDNN), root mean square of successive differences between heartbeats (RMSSD), low frequency (LF), and high frequency (HF).

Figure 2 denotes the agreement for the PPG waveforms compared to the ECG waveforms. Minimal difference was noted in heart rate (~0.01%); however, this was sufficient to raise PRV SDNN by ~1% and PRV RMSSD by ~3–6% (Figure 2). Relative HF was higher when derived with PRV (~1–2%), while both relative LF (~4–8%) and LF/HF (~10–40%) were lower (Figure 3). Effect size differences between HRV and PRV metrics are shown in Figure 3, with negligible Cohen’s d values for heart rate and SDNN (absolute Cohen’s d < 0.11). However, RMSSD, relative LF, relative HF, and LF/HF had small effect sizes (absolute Cohen’s *d* range: 0.20–0.35) (Figure 3).

### 3.2. Sampling Frequency Validity for Heart Rate Variability vs. Pulse Rate Variability Metrics

**Figure 4:**
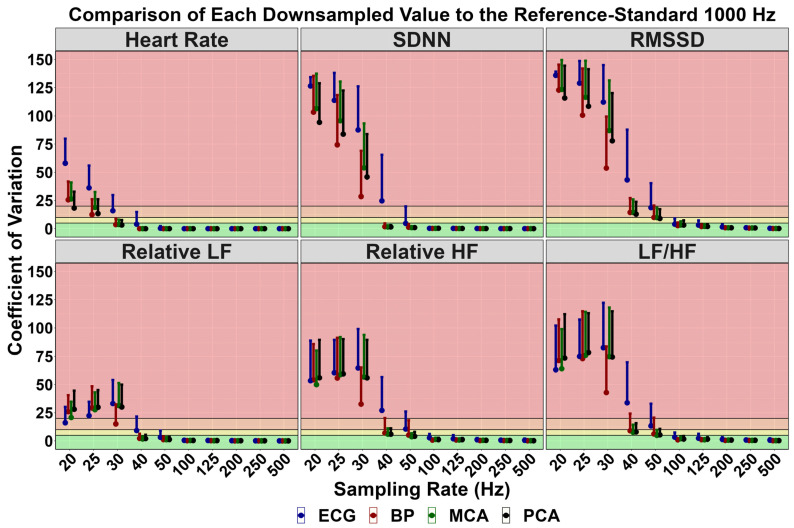
Coefficient of variation values comparing the validity of downsampled heart rate variability and pulse rate variability metrics to a “*reference-standard*” 1000 Hz recording in 54 individuals (29 females and 25 males). Heart rate variability metrics were obtained from an electrocardiography (ECG) and pulse rate variability metrics from three devices using photoplethysmography (PPG) measuring blood pressure (BP) at the finger and velocity within the middle cerebral artery (MCA) and posterior cerebral artery (PCA). Thresholds of <5% (excellent), 5–10% (good), 10–20% (acceptable), and >20% (unacceptable) are shown with green, yellow, orange, and red shading. Standard deviation of N-N intervals (SDNN), root mean square of successive differences between heartbeats (RMSSD), relative low frequency (LF), and relative high frequency (HF).

**Figure 5:**
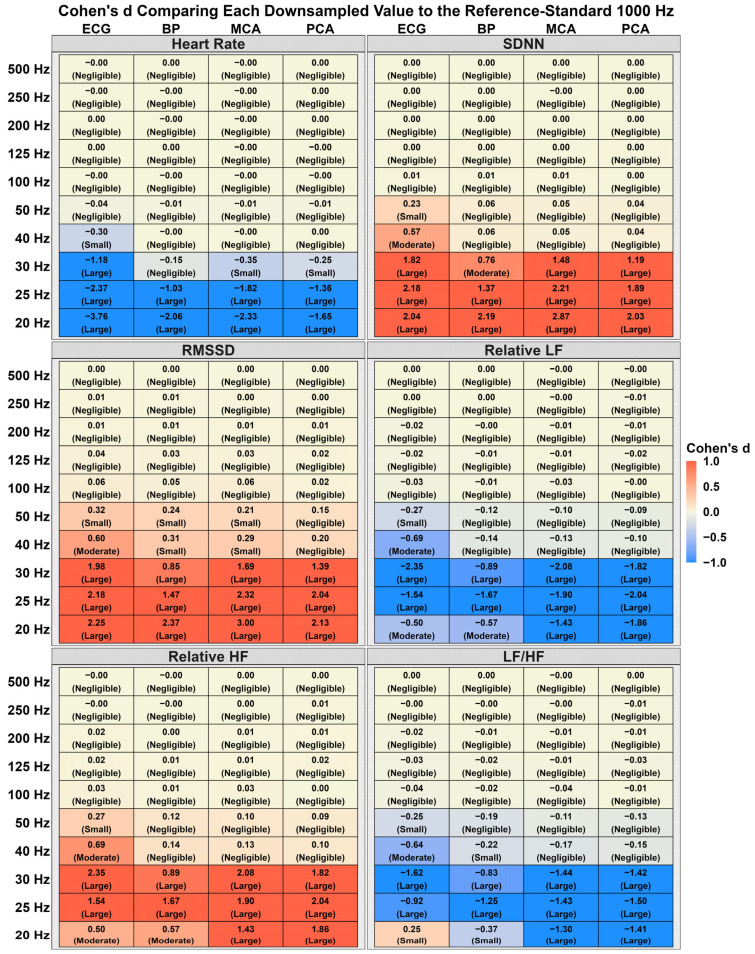
Cohen’s *d* effect size values comparing the validity of downsampled heart rate variability and pulse rate variability metrics to a “*reference-standard*” 1000 Hz recording in 54 individuals (29 females and 25 males). Heart rate variability metrics were obtained from an electrocardiography (ECG) and pulse rate variability metrics from three devices using photoplethysmography (PPG) measuring blood pressure (BP) at the finger and velocity within the middle cerebral artery (MCA) and posterior cerebral artery (PCA). Cohen’s *d* effect sizes determined the magnitude difference between comparisons with thresholds of <0.20 (negligible), 0.20–0.50 (small), 0.50–0.80 (moderate), and >0.80 (large). A negative value (blue) corresponds to a sampling frequency that produced a higher value than the 1000 Hz recording, while a positive value (red) corresponds to a sampling frequency that produced a lower value than the 1000 Hz recording. Standard deviation of N-N intervals (SDNN), root mean square of successive differences between heartbeats (RMSSD), low frequency (LF), and high frequency (HF).

Table 1 displays the mean and standard deviation for HRV/PRV metrics at all sampling frequencies. While homogeneous at higher sampling frequencies, PRV parameters begin to diverge ~1–5% at lower sampling frequencies (~40–100 Hz). However, the linear regressions in Table 2 denoted these became statistically different at ~25–30 Hz. Bland–Altman plots with the associated 95% LOA are displayed in Supplemental Figures S1–S6, comparing the HRV/PRV values obtained from each downsampled frequency. Further, CoV and Cohen’s *d* effect sizes for these comparisons are additionally detailed in Figure 4 and Figure 5, respectively.

For ECG, the lowest sampling rate with valid metrics was as follows: heart rate (50 Hz; mean bias: −0.00% [95% LOA: −0.02–0.02%]; CoV: 0.34%), SDNN (100 Hz; mean bias: 0.30% [95% LOA: −0.92–1.51%]; CoV: 0.69%), RMSSD (200 Hz; mean bias: 1.50% [95% LOA: −5.57–8.57]; CoV: 1.53%), relative LF (100 Hz; mean bias: −0.54% [95% LOA: −3.59–2.50%]; CoV: 0.58%), relative HF (200 Hz; mean bias: 0.94% [95% LOA: −3.84–5.72%]; CoV: 1.09%), and LF/HF (200 Hz; mean bias: −1.23% [95% LOA: −7.36–4.91%]; CoV: 1.37%) (Supplemental Figures S1–S6). The linear regressions noted HRV parameters were similar to 1000 Hz until a sampling rate of 50 Hz, aside from RMSSD which required a sampling rate of 100 Hz (Table 2).

For the BP PPG, the lowest sampling rate with valid metrics was as follows: heart rate (40 Hz; mean bias: −0.00% [95% LOA: −0.12–0.10%]; CoV: 0.01%), SDNN (100 Hz; mean bias: 0.31% [95% LOA: −0.34–0.97%]; CoV: 0.22%), RMSSD (250 Hz; mean bias: 0.68% [95% LOA: −0.95–2.31%]; CoV: 0.84%), relative LF (100 Hz; mean bias: −0.27% [95% LOA: −1.50–0.96%]; CoV: 0.26%), relative HF (100 Hz; mean bias: 0.86% [95% LOA: −2.29–4.01%]; CoV: 0.81%), and LF/HF (100 Hz; mean bias: −1.13% [95% LOA: −5.07–2.81%]; CoV: 1.07%) (Supplemental Figures S1–S6). The linear regressions noted BP PRV parameters were similar to 1000 Hz until a sampling rate of 30 Hz (Table 2).

For the MCA PPG, the lowest sampling rate with valid metrics was as follows: heart rate (40 Hz; mean bias: −0.00% [95% LOA: −0.01–0.01%]; CoV: 0.01%), SDNN (50 Hz; mean bias: 1.32% [95% LOA: −1.11–3.75%]; CoV: 0.93%), RMSSD (200 Hz; mean bias: 0.71% [95% LOA: −1.56–2.98%]; CoV: 0.81%), relative LF (100 Hz; mean bias: −0.57% [95% LOA: −2.73–1.59%]; CoV: 0.45%), relative HF (100 Hz; mean bias: 1.78% [95% LOA: −2.20–5.76%]; CoV: 1.46%), and LF/HF (100 Hz; mean bias: −2.35% [95% LOA: −7.78–3.07%]; CoV: 1.91%) (Supplemental Figures S1–S6). The linear regressions noted MCA PRV parameters were similar to 1000 Hz until a sampling rate of 30 Hz (Table 2).

For the PCA PPG, the lowest sampling rate with valid metrics was as follows: heart rate (40 Hz; mean bias: −0.00% [95% LOA: −0.01–0.01%]; CoV: 0.01%), SDNN (50 Hz; mean bias: 1.23% [95% LOA: −1.74–4.20%]; CoV: 0.99%), RMSSD (200 Hz; mean bias: 0.66% [95% LOA: −2.03–3.35%]; CoV: 0.81%), relative LF (100 Hz; mean bias: 0.03% [95% LOA: −3.80–3.85%]; CoV: 0.44%), relative HF (100 Hz; mean bias: 0.46% [95% LOA: −4.88–5.81%]; CoV: 1.20%), and LF/HF (100 Hz; mean bias: −0.44% [95% LOA: −9.26– 8.38%]; CoV: 1.63%) (Supplemental Figures S1–S6). The linear regressions noted MCA PRV parameters were similar to 1000 Hz until a sampling rate of 30 Hz (Table 2).

## 4. Discussion

To help guide future studies employing PPG wearables/sensors, this investigation sought to explore the relationship between HRV and PRV at different anatomic locations and elucidate the minimal sampling rate required to produce valid PRV metrics. The main findings were as follows: (1) sample rate had a greater impact on HRV metrics compared to PRV, where the latter displayed greater validity at slightly lower sampling rates; (2) anatomical location had a mild impact on PRV parameters, albeit this became more pronounced with a lower sampling rate; (3) PRV-derived metrics had a small, not significant bias towards producing slightly greater time- and frequency-domain metrics compared to HRV-derived metrics, although this fell within normal limits at 1000 Hz. The collective findings are in agreement with past reports that HRV and PRV are not surrogates but, rather, are individual biomarkers [13,14]. Future work utilizing smartwatches and/or neuroimaging equipment may benefit from employing PRV analysis. Nonetheless, researchers need to consider the sampling rate of their technology to understand the degree of variability they can expect in their measures.

### 4.1. Comparison to Previous Research and Physiological Underpinnings

The current findings are in agreement with several previous investigations highlighting HRV derived from an “R-spike” is not a direct surrogate of PRV derived from a pulsatile waveform peak [13,17]. As described in the introduction, the “R-spike” of the QRS complex lasts a maximum of 20 milliseconds [12], whereas the pulsatile waveform peak exists over 30–40 milliseconds [11]. Ultimately, this underpins Supplemental Figures S1–S6, demonstrating tighter limits of agreement for PRV-derived estimates at lower sampling rates. For example, the mean difference in absolute heart rate from an ECG was 0.0%, −0.5%, and −5.7% at sampling rates of 100, 50, and 40 Hz, respectively. Conversely, the PPG mean difference remained at ~0.0% at the same frequencies (Supplemental Figure S1). This translated to greater validity at lower sampling rates. Moreover, if using a validity threshold of <5% for the 95% LOA and CoV measurements, a PPG sampling rate of ~100–200 Hz would be required to satisfy these criteria (Figure 4 and Figure 5 and Supplemental Figures S1–S6). Nevertheless, other investigations have noted physiological thresholds of ~20% variation being sufficient to elicit an acceptable level of robustness in physiological measures. If using this threshold, a sample rate of ~40–50 Hz would be required for PRV metrics, which is homogeneous with the linear regression analysis, noting these produced statistically similar estimates (Table 2). Further, Table 2 displays the group averages for the downsampled time- and frequency-domain PRV estimates. At 40 Hz, all group means were within two units of the 1000 Hz reference-standard estimates, excluding RMSSD, which was ~3–6 milliseconds different. Therefore, while the group estimates were similar at 40 Hz, researchers using wearable technology and/or neuroimaging equipment at a sampling rate lower than 100 Hz to demarcate clinical or group differences must consider the within-measure variability demonstrated in the current investigation. Therefore, a study examining group differences can utilize a sampling rate of 40 Hz; however, if an investigation is studying longitudinal changes within the same participants, a sampling rate of 100 Hz or greater is recommended. Finally, the Nyquist theorem states that to accurately obtain meaningful information from a waveform signal, the sampling rate must be double the highest frequency present in the signal [52]. With a maximal heart rate of 200 beats per minute, a sampling frequency of ~7 Hz would be sufficient to determine absolute heart rate [53]. The current findings denoted this was not the case within the current study; however, this was likely due to the given downsampled waveform not producing sufficient data points to obtain a reliable QRS spike or systolic peak waveform [53]. Therefore, absolute heart rate would be computable with simpler cyclical calculations that are not designed to detect millisecond differences.

Anatomical location also appeared to have an influence on PRV metrics, which is homogenous to previous investigations [16,17]. For example, Wong et al. [16] demonstrated PRV differed between fingers on the left and right hand, while Yuda et al. [17] found PRV was different when measured between the wrist and the fingers on the same arm. This investigation expanded on these previous studies by including pulsatile waveforms from both the cerebral and peripheral vasculature. The cerebrovasculature receives direct innervation from both sympathetic and parasympathetic nervous system fibers, while the peripheral vasculature is only innervated by sympathetic fibers [21]. Due to this greater autonomic regulation of the cerebrovasculature, lower variation would be expected in PRV metrics, as both branches allow for more precise regulation to ensure cerebral blood flow remains homeostatic [21]. Minimal deviation was noted for PRV between anatomical differences at higher sampling rates (≥40 Hz); however, a greater mean difference was noted for the MCA and PCA waveforms compared to the blood pressure below this threshold (Supplemental Figures S1–S6). Finally, it is important to consider the peripheral circulation is not a simple, passive conduit for blood, but rather serves to redirect blood flow to working skeletal muscles. Therefore, differences between peripheral locations would be expected based on the conditions where data are collected (e.g., supine vs. upright). This would become more prominent during real-life recordings with wearable technology.

Lastly, previous reports have demonstrated HRV and PRV are not entirely homogeneous biomarkers [13,17]. The current findings are in agreement with this as a small bias was found, albeit insignificant; however, this did appear to be somewhat metric-dependent. For example, while no bias existed for absolute heart rate (~0.01%), bias was seen for frequency-domain PRV metrics (mean difference range: ~1–40%; small effect sizes) compared to time-domain (mean difference range: ~1–6%; negligible-to-small effect size) (Figure 2 and Figure 3). The greatest difference was found in the LF/HF ratio (Figure 2 and Figure 3), which parallels the findings by Yuda and colleagues [17]. Further, it is imperative to note the HRV and PRV comparisons were completed at 1000 Hz, and greater discrepancies may be noted at lower sampling rates. The physiological explanation for this is due to PRV indirectly measuring each left ventricular contraction downstream where numerous factors can introduce variations in transient time to an anatomical location of interest [20,54,55]. For example, Sung and colleagues [20] collected arterial Doppler waveform data from a phantom, finding the spectral Doppler traces were modified based on resistance, compliance, and pulse rate. Further, Lefferts et al. [54] identified older age, carotid pulse pressure, large arterial stiffening, and forward wave energy influence the pulsatile waveform. While some of these factors are non-modifiable, vessel diameter has also been linked to influencing the pulsatile waveform [55], which can change on a beat-to-beat basis [56]. These factors can lead to millisecond differences when measuring blood volumetric changes via PPG. While this may seem inconsequential, Figure 4 and Figure 5 demonstrate RMSSD was the most sensitive metric, as it displayed the greatest bias at 250 Hz compared to the other measures. A sampling rate of 250 Hz, would correspond to an “R-spike” deviation of only 4 milliseconds, demonstrating why the PPG transient time delay would artificially inflate the quantification of parasympathetic nervous system activity [57].

### 4.2. Future Directions and Limitations

While the HRV and PRV comparisons were completed at 1000 Hz, the vast majority of all wearable PPG devices have a sampling rate lower than 100 Hz. Therefore, the results of the current investigation demonstrate it is imperative these devices are capable of capturing high-quality data with a clear systolic pulsatile peak. As an illustration, previous examples of transcranial Doppler ultrasound waveforms have been collected where the systolic peak is clipped [58] or incorrectly captured [59]. Supplemental Figures S7 and S8 demonstrate the impact of these data being collected at various sampling rates. These highlights that researchers must be cognizant of the quality of the pulsatile waveforms being collected from the utilized wearable technology and/or neuroimaging equipment. For example, Supplemental Figure S8 demonstrates a pulsatile waveform where the systolic peak is not fully captured. This results in a ~40–50 millisecond plateau where the P-waveform peak could be captured, thus skewing PRV estimates. 

A limitation of the current investigation is that only younger participants were included in the current analyses. As stated above, the nature of pulsatile waveforms is influenced by aging and arterial stiffness [54], where the latter has been demonstrated to be associated with numerous clinical presentations [60]. Replication of these findings in older and clinical samples will be fruitful to understand if the sampling frequencies found in the current investigation are externally valid to the wider population. Nevertheless, the lowest sampling rate expected would be within young, healthy adults, and thus, these results provide a foundation future research can build on. The data utilized in this investigation were free of ectopic beats or any other arrhythmias. Data containing misaligned beats, ectopic beats, or other arrhythmias would likely require a higher sampling rate to be able to discern these robustly. Hence, it is likely clinical populations may require a slightly higher sampling frequency.

A final limitation of the current methodological approach is that ECG and PPG recordings were taken at 1000 Hz and downsampled to various frequencies, rather than obtaining simultaneous recordings from different devices concurrently. However, completing data collection with devices at various sampling rates may not be feasible, especially if one wished to complete a recording at all 11 required durations of interest.

## 5. Conclusions

The current findings provide further evidence that PRV and HRV are distinct biomarkers. With this in mind, a primary benefit of PRV is that it allows one to obtain information on the autonomic nervous system under real-life conditions based on the rapid expansion of wearable technology and the commercialization of neuroimaging equipment. Further, due to the longer-lived nature of the systolic pulsatile waveform compared to the QRS complex, PRV parameters are capable of being deduced at lower sampling rates compared to time- and frequency-domain estimates obtained from short-term HRV recordings. For highly robust measures, a sampling rate of 100 Hz is recommended; however, a sampling rate of 40 Hz produced acceptable validity based on standards used in previous literature. Moreover, researchers using a lower sampling rate must consider variability due to insufficient sampling rates when comparing groups and/or clinical populations, which may be further exacerbated when measured outside of controlled laboratory conditions.

## Figures and Tables

**Table 1:** Mean and standard deviation heart rate variability metrics from an electrocardiography (ECG) and pulse rate variability metrics from three devices using photoplethysmography measuring blood pressure (BP) at the finger and velocity within the middle cerebral artery (MCA) and posterior cerebral artery (PCA). Data were collected in 54 individuals (29 females and 25 males) and downsampled to various sampling rates.

Variable	Sampling Rate	ECG	BP	MCA	PCA
Heart rate (beats per minutes)	1000	80.0 (10.9)	80.0 (10.9)	80.0 (10.9)	80.0 (10.9)
	500	80.0 (10.9)	80.0 (10.9)	80.0 (10.9)	80.0 (10.9)
	250	80.0 (10.9)	80.0 (10.9)	80.0 (10.9)	80.0 (10.9)
	200	80.0 (10.9)	80.0 (10.9)	80.0 (10.9)	80.0 (10.9)
	125	80.0 (10.9)	80.0 (10.9)	80.0 (10.9)	80.0 (10.9)
	100	80.0 (10.9)	80.0 (10.9)	80.0 (10.9)	80.0 (10.9)
	50	79.5 (11.1)	79.8 (10.9)	79.8 (10.9)	79.8 (10.9)
	40	76.2 (14.0)	80.0 (10.9)	80.0 (10.9)	80.0 (10.9)
	30	65.0 (14.2)	77.4 (9.3)	78.2 (12.3)	76.5 (8.6)
	25	48.7 (15.2)	66.0 (9.6)	67.6 (13.1)	61.2 (9.7)
	20	34.6 (13.1)	61.9 (11.0)	56.0 (12.4)	55.0 (10.6)
SDNN (milliseconds)	1000	61.1 (22.1)	63.5 (22.6)	63.4 (22.5)	62.7 (21.7)
	500	61.1 (22.1)	63.5 (22.6)	63.4 (22.6)	62.8 (21.7)
	250	61.1 (22.1)	63.5 (22.5)	63.5 (22.5)	62.7 (21.6)
	200	61.1 (22.2)	63.6 (22.6)	63.5 (22.5)	62.8 (21.6)
	125	61.2 (22.0)	63.6 (22.4)	63.6 (22.5)	62.8 (21.6)
	100	61.3 (22.2)	63.6 (22.4)	63.6 (22.5)	62.9 (21.6)
	50	70.5 (51.9)	64.4 (22.2)	64.8 (22.8)	63.8 (21.6)
	40	145.2 (209.4)	64.3 (22.0)	64.8 (22.5)	63.8 (21.3)
	30	396.7 (259.4)	154.7 (106.2)	141.5 (143.7)	174.7 (104.6)
	25	804.8 (482.3)	371.2 (228.8)	352.4 (297.2)	483.0 (268.2)
	20	1441.7 (955.9)	472.3 (284.1)	618.9 (358.5)	633.3 (280.5)
RMSSD (milliseconds)	1000	29.3 (17.6)	36.6 (23.8)	33.6 (18.7)	32.8 (19.2)
	500	29.3 (17.6)	36.6 (23.8)	33.6 (18.7)	32.9 (19.2)
	250	29.4 (17.6)	36.7 (23.7)	33.7 (18.6)	32.9 (18.8)
	200	29.5 (17.6)	36.9 (23.9)	33.8 (18.6)	33.0 (19.2)
	125	29.9 (17.2)	37.2 (23.4)	34.2 (18.5)	33.4 (18.7)
	100	30.4 (17.7)	37.1 (22.7)	34.5 (18.4)	33.9 (19.1)
	50	46.1 (73.2)	40.1 (22.1)	38.0 (18.2)	36.8 (18.6)
	40	151.2 (288.1)	41.0 (21.3)	39.3 (17.6)	38.2 (17.4)
	30	514.5 (345.8)	194.6 (158.9)	165.4 (219.1)	225.4 (159.5)
	25	1064.7 (672.2)	510.3 (328.1)	480.6 (430.9)	670.4 (387.9)
	20	1872.1 (1159.4)	661.2 (414.8)	858.0 (491.2)	877.3 (398.2)
Relative LF (normalized units)	1000	81.8 (11.0)	77.4 (13.8)	77.6 (12.6)	78.8 (12.4)
	500	81.8 (11.0)	77.4 (13.8)	77.6 (12.5)	78.8 (12.4)
	250	81.8 (10.9)	77.3 (13.9)	77.7 (12.5)	78.8 (12.4)
	200	81.6 (11.2)	77.3 (13.9)	77.6 (12.6)	78.7 (12.5)
	125	81.5 (11.2)	77.1 (13.9)	77.5 (12.5)	78.7 (12.5)
	100	81.4 (11.2)	77.4 (13.6)	77.5 (12.7)	78.5 (12.7)
	50	78.5 (12.9)	76.2 (14.2)	76.2 (12.6)	77.5 (13.2)
	40	72.8 (14.8)	76.1 (14.1)	75.8 (13.3)	77.2 (13.2)
	30	51.7 (14.4)	51.8 (14.4)	64.5 (16.7)	50.7 (14.5)
	25	63.5 (12.8)	51.8 (11.3)	54.4 (15.1)	55.1 (12.6)
	20	74.8 (16.4)	53.1 (12.3)	56.6 (12.7)	61.2 (12.3)
Relative HF (normalized units)	1000	18.2 (11.0)	22.6 (13.8)	22.4 (12.6)	21.2 (12.4)
	500	18.2 (11.0)	22.6 (13.8)	22.4 (12.5)	21.2 (12.4)
	250	18.2 (10.9)	22.7 (13.9)	22.3 (12.5)	21.2 (12.4)
	200	18.4 (11.2)	22.7 (13.9)	22.4 (12.6)	21.3 (12.5)
	125	18.5 (11.2)	22.9 (13.9)	22.5 (12.5)	21.3 (12.5)
	100	18.6 (11.2)	22.6 (13.6)	22.5 (12.7)	21.5 (12.7)
	50	21.5 (12.9)	23.8 (14.2)	23.8 (12.6)	22.5 (13.2)
	40	27.2 (14.8)	23.9 (14.1)	24.2 (13.3)	22.8 (13.2)
	30	48.3 (14.4)	48.2 (14.4)	35.5 (16.7)	49.3 (14.5)
	25	36.5 (12.8)	48.2 (11.3)	45.6 (15.1)	44.9 (12.6)
	20	25.2 (16.4)	46.9 (12.3)	43.4 (12.7)	38.8 (12.3)
LF/HF (percent)	1000	6.5 (4.3)	5.2 (3.7)	5.1 (3.7)	5.5 (3.8)
	500	6.5 (4.3)	5.2 (3.7)	5.1 (3.7)	5.5 (3.8)
	250	6.5 (4.3)	5.2 (3.7)	5.1 (3.7)	5.5 (3.7)
	200	6.4 (4.3)	5.2 (3.6)	5.1 (3.7)	5.5 (3.7)
	125	6.4 (4.2)	5.1 (3.6)	5.1 (3.6)	5.4 (3.7)
	100	6.3 (4.1)	5.2 (3.7)	5.1 (3.7)	5.4 (3.6)
	50	5.5 (3.9)	4.8 (3.3)	4.5 (2.9)	5.1 (3.4)
	40	4.1 (3.3)	4.7 (3.2)	4.4 (2.8)	4.9 (3.2)
	30	1.4 (1.2)	1.4 (1.1)	2.6 (2.0)	1.4 (1.4)
	25	2.7 (4.0)	1.2 (0.8)	1.6 (1.5)	1.5 (1.2)
	20	10.4 (21.7)	1.4 (1.2)	1.7 (1.9)	1.9 (1.1)

**Table 2:** Linear regression output for all transfer function analysis variables in 54 participants (29 females and 24 males) comparing each downsampled waveform to each “*reference-standard*” 1000 Hz recording.

Variable	Frequency	ECG	BP	MCA	PCA
Heart rate (beats per minutes)	Intercept	4.37 (95% CI: 4.31, 4.43); *p* < 0.001	4.37 (95% CI: 4.33, 4.41); *p* < 0.001	4.37 (95% CI: 4.33, 4.41); *p* < 0.001	4.37 (95% CI: 4.34, 4.41); *p* < 0.001
	500 Hz	−0.00 (95% CI: −0.08, 0.08); *p* > 0.999	0.00 (95% CI: −0.06, 0.06); *p* > 0.999	−0.00 (95% CI: −0.05, 0.05); *p* > 0.999	0.00 (95% CI: −0.05, 0.05); *p* > 0.999
	250 Hz	−0.00 (95% CI: −0.08, 0.08); *p* > 0.999	−0.00 (95% CI: −0.06, 0.06); *p* > 0.999	−0.00 (95% CI: −0.05, 0.05); *p* > 0.999	0.00 (95% CI: −0.05, 0.05); *p* > 0.999
	200 Hz	0.00 (95% CI: −0.08, 0.08); *p* = 0.999	−0.00 (95% CI: −0.06, 0.06); *p* > 0.999	−0.00 (95% CI: −0.05, 0.05); *p* > 0.999	0.00 (95% CI: −0.05, 0.05); *p* > 0.999
	125 Hz	0.00 (95% CI: −0.08, 0.08); *p* > 0.999	0.00 (95% CI: −0.06, 0.06); *p* > 0.999	−0.00 (95% CI: −0.05, 0.05); *p* > 0.999	−0.00 (95% CI: −0.05, 0.05); *p* > 0.999
	100 Hz	−0.00 (95% CI: −0.08, 0.08); *p* > 0.999	−0.00 (95% CI: −0.06, 0.06); *p* > 0.999	−0.00 (95% CI: −0.05, 0.05); *p* > 0.999	−0.00 (95% CI: −0.05, 0.05); *p* > 0.999
	50 Hz	−0.01 (95% CI: −0.09, 0.08); *p* = 0.875	−0.00 (95% CI: −0.06, 0.06); *p* = 0.945	−0.00 (95% CI: −0.06, 0.05); *p* = 0.941	−0.00 (95% CI: −0.06, 0.05); *p* = 0.941
	40 Hz	−0.06 (95% CI: −0.14, 0.02); *p* = 0.167	−0.00 (95% CI: −0.06, 0.06); *p* = 0.999	−0.00 (95% CI: −0.05, 0.05); *p* > 0.999	−0.00 (95% CI: −0.05, 0.05); *p* > 0.999
	30 Hz	**−0.22 (95% CI: −0.31, −0.14); *p* < 0.001**	−0.03 (95% CI: −0.08, 0.03); *p* = 0.404	−0.04 (95% CI: −0.10, 0.01); *p* = 0.134	−0.03 (95% CI: −0.08, 0.02); *p* = 0.263
	25 Hz	**−0.54 (95% CI: −0.62, −0.46); *p* < 0.001**	**−0.18 (95% CI: −0.24, −0.12); *p* < 0.001**	**−0.27 (95% CI: −0.33, −0.22); *p* < 0.001**	**−0.19 (95% CI: −0.25, −0.14); *p* < 0.001**
	20 Hz	**−0.91 (95% CI: −1.00, −0.83); *p* < 0.001**	**−0.37 (95% CI: −0.43, −0.31); *p* < 0.001**	**−0.38 (95% CI: −0.44, −0.33); *p* < 0.001**	**−0.26 (95% CI: −0.32, −0.21); *p* < 0.001**
SDNN (milliseconds)	Intercept	4.04 (95% CI: 3.90, 4.19); *p* < 0.001	4.08 (95% CI: 3.94, 4.23); *p* < 0.001	4.08 (95% CI: 3.95, 4.20); *p* < 0.001	4.08 (95% CI: 3.95, 4.22); *p* < 0.001
	500 Hz	0.00 (95% CI: −0.20, 0.21); *p* = 0.999	0.00 (95% CI: −0.21, 0.21); *p* > 0.999	0.00 (95% CI: −0.18, 0.18); *p* = 0.999	0.00 (95% CI: −0.19, 0.19); *p* = 0.998
	250 Hz	0.00 (95% CI: −0.20, 0.21); *p* = 0.998	0.00 (95% CI: −0.21, 0.21); *p* = 0.997	0.00 (95% CI: −0.18, 0.18); *p* = 0.998	0.00 (95% CI: −0.19, 0.19); *p* = 0.997
	200 Hz	0.00 (95% CI: −0.20, 0.21); *p* = 0.995	0.00 (95% CI: −0.21, 0.21); *p* = 0.994	0.00 (95% CI: −0.18, 0.18); *p* = 0.992	0.00 (95% CI: −0.18, 0.19); *p* = 0.990
	125 Hz	0.00 (95% CI: −0.20, 0.21); *p* = 0.979	0.00 (95% CI: −0.20, 0.21); *p* = 0.983	0.00 (95% CI: −0.18, 0.18); *p* = 0.986	0.00 (95% CI: −0.18, 0.19); *p* = 0.981
	100 Hz	0.00 (95% CI: −0.20, 0.21); *p* = 0.977	0.00 (95% CI: −0.20, 0.21); *p* = 0.976	0.00 (95% CI: −0.18, 0.18); *p* = 0.968	0.00 (95% CI: −0.18, 0.19); *p* = 0.978
	50 Hz	0.08 (95% CI: −0.13, 0.29); *p* = 0.447	0.02 (95% CI: −0.18, 0.23); *p* = 0.827	0.02 (95% CI: −0.16, 0.20); *p* = 0.838	0.02 (95% CI: −0.17, 0.21); *p* = 0.848
	40 Hz	**0.46 (95% CI: 0.26, 0.67); *p* < 0.001**	0.02 (95% CI: −0.18, 0.23); *p* = 0.814	0.02 (95% CI: −0.16, 0.20); *p* = 0.817	0.02 (95% CI: −0.17, 0.20); *p* = 0.844
	30 Hz	**1.68 (95% CI: 1.48, 1.89); *p* < 0.001**	**0.50 (95% CI: 0.29, 0.70); *p* < 0.001**	**0.91 (95% CI: 0.73, 1.09); *p* < 0.001**	**0.76 (95% CI: 0.58, 0.95); *p* < 0.001**
	25 Hz	**2.46 (95% CI: 2.26, 2.67); *p* < 0.001**	**1.41 (95% CI: 1.20, 1.61); *p* < 0.001**	**1.89 (95% CI: 1.71, 2.08); *p* < 0.001**	**1.59 (95% CI: 1.41, 1.78); *p* < 0.001**
	20 Hz	**3.07 (95% CI: 2.87, 3.28); *p* < 0.001**	**2.12 (95% CI: 1.91, 2.33); *p* < 0.001**	**2.21 (95% CI: 2.03, 2.39); *p* < 0.001**	**1.84 (95% CI: 1.66, 2.03); *p* < 0.001**
RMSSD (milliseconds)	Intercept	3.24 (95% CI: 3.06, 3.42); *p* < 0.001	3.40 (95% CI: 3.22, 3.57); *p* < 0.001	3.37 (95% CI: 3.21, 3.52); *p* < 0.001	3.45 (95% CI: 3.29, 3.61); *p* < 0.001
	500 Hz	0.00 (95% CI: −0.25, 0.26); *p* = 0.988	0.00 (95% CI: −0.25, 0.25); *p* = 0.995	0.00 (95% CI: −0.22, 0.22); *p* = 0.989	0.00 (95% CI: −0.23, 0.23); *p* = 0.989
	250 Hz	0.01 (95% CI: −0.25, 0.26); *p* = 0.951	0.01 (95% CI: −0.24, 0.26); *p* = 0.958	0.01 (95% CI: −0.21, 0.22); *p* = 0.949	0.01 (95% CI: −0.22, 0.23); *p* = 0.955
	200 Hz	0.02 (95% CI: −0.24, 0.27); *p* = 0.908	0.01 (95% CI: −0.24, 0.26); *p* = 0.930	0.01 (95% CI: −0.21, 0.23); *p* = 0.926	0.01 (95% CI: −0.22, 0.24); *p* = 0.922
	125 Hz	0.04 (95% CI: −0.21, 0.29); *p* = 0.753	0.03 (95% CI: −0.22, 0.28); *p* = 0.834	0.03 (95% CI: −0.19, 0.24); *p* = 0.804	0.03 (95% CI: −0.20, 0.25); *p* = 0.824
	100 Hz	0.06 (95% CI: −0.20, 0.31); *p* = 0.670	0.04 (95% CI: −0.21, 0.29); *p* = 0.764	0.05 (95% CI: −0.17, 0.26); *p* = 0.677	0.04 (95% CI: −0.19, 0.26); *p* = 0.760
	50 Hz	**0.30 (95% CI: 0.05, 0.56); *p* = 0.021**	0.16 (95% CI: −0.09, 0.41); *p* = 0.219	0.16 (95% CI: −0.06, 0.37); *p* = 0.162	0.14 (95% CI: −0.09, 0.37); *p* = 0.233
	40 Hz	**0.88 (95% CI: 0.63, 1.13); *p* < 0.001**	0.20 (95% CI: −0.05, 0.46); *p* = 0.110	0.21 (95% CI: −0.01, 0.42); *p* = 0.060	0.17 (95% CI: −0.05, 0.40); *p* = 0.133
	30 Hz	**2.65 (95% CI: 2.40, 2.91); *p* < 0.001**	**1.03 (95% CI: 0.78, 1.28); *p* < 0.001**	**1.76 (95% CI: 1.54, 1.98); *p* < 0.001**	**1.51 (95% CI: 1.28, 1.73); *p* < 0.001**
	25 Hz	**3.52 (95% CI: 3.26, 3.77); *p* < 0.001**	**2.28 (95% CI: 2.03, 2.53); *p* < 0.001**	**2.89 (95% CI: 2.67, 3.11); *p* < 0.001**	**2.50 (95% CI: 2.27, 2.73); *p* < 0.001**
	20 Hz	**4.14 (95% CI: 3.89, 4.40); *p* < 0.001**	**3.11 (95% CI: 2.86, 3.36); *p* < 0.001**	**3.22 (95% CI: 3.01, 3.44); *p* < 0.001**	**2.78 (95% CI: 2.56, 3.01); *p* < 0.001**
Relative LF (normalized units)	Intercept	4.39 (95% CI: 4.34, 4.45); *p* < 0.001	4.34 (95% CI: 4.28, 4.39); *p* < 0.001	4.35 (95% CI: 4.30, 4.41); *p* < 0.001	4.33 (95% CI: 4.27, 4.39); *p* < 0.001
	500 Hz	−0.00 (95% CI: −0.07, 0.07); *p* = 0.999	0.00 (95% CI: −0.08, 0.08); *p* = 0.995	−0.00 (95% CI: −0.08, 0.07); *p* = 0.989	−0.00 (95% CI: −0.08, 0.08); *p* = 0.988
	250 Hz	0.00 (95% CI: −0.07, 0.07); *p* = 0.987	0.00 (95% CI: −0.08, 0.08); *p* = 0.988	−0.00 (95% CI: −0.07, 0.07); *p* = 0.994	−0.00 (95% CI: −0.08, 0.08); *p* = 0.964
	200 Hz	−0.00 (95% CI: −0.08, 0.07); *p* = 0.938	−0.00 (95% CI: −0.08, 0.08); *p* = 0.988	−0.00 (95% CI: −0.08, 0.07); *p* = 0.973	−0.00 (95% CI: −0.08, 0.08); *p* = 0.951
	125 Hz	−0.00 (95% CI: −0.08, 0.07); *p* = 0.916	−0.00 (95% CI: −0.08, 0.08); *p* = 0.962	−0.00 (95% CI: −0.08, 0.07); *p* = 0.964	−0.00 (95% CI: −0.09, 0.08); *p* = 0.909
	100 Hz	−0.01 (95% CI: −0.08, 0.07); *p* = 0.884	−0.00 (95% CI: −0.08, 0.08); *p* = 0.946	−0.01 (95% CI: −0.08, 0.07); *p* = 0.880	0.00 (95% CI: −0.08, 0.08); *p* = 0.995
	50 Hz	−0.05 (95% CI: −0.12, 0.03); *p* = 0.218	−0.02 (95% CI: −0.10, 0.06); *p* = 0.625	−0.02 (95% CI: −0.09, 0.06); *p* = 0.620	−0.02 (95% CI: −0.10, 0.06); *p* = 0.656
	40 Hz	**−0.13 (95% CI: −0.20, −0.06); *p* = 0.001**	−0.03 (95% CI: −0.11, 0.05); *p* = 0.497	−0.02 (95% CI: −0.10, 0.05); *p* = 0.523	−0.02 (95% CI: −0.10, 0.06); *p* = 0.637
	30 Hz	**−0.49 (95% CI: −0.56, −0.42); *p* < 0.001**	**−0.21 (95% CI: −0.29, −0.13); *p* < 0.001**	**−0.46 (95% CI: −0.54, −0.39); *p* < 0.001**	**−0.42 (95% CI: −0.51, −0.34); *p* < 0.001**
	25 Hz	**−0.26 (95% CI: −0.34, −0.19); *p* < 0.001**	**−0.38 (95% CI: −0.46, −0.30); *p* < 0.001**	**−0.37 (95% CI: −0.44, −0.29); *p* < 0.001**	**−0.41 (95% CI: −0.49, −0.32); *p* < 0.001**
	20 Hz	**−0.11 (95% CI: −0.18, −0.03); *p* = 0.005**	**−0.32 (95% CI: −0.40, −0.25); *p* < 0.001**	**−0.26 (95% CI: −0.34, −0.19); *p* < 0.001**	**−0.38 (95% CI: −0.47, −0.30); *p* < 0.001**
Relative HF (normalized units)	Intercept	2.74 (95% CI: 2.58, 2.90); *p* < 0.001	2.95 (95% CI: 2.81, 3.10); *p* < 0.001	2.89 (95% CI: 2.75, 3.03); *p* < 0.001	2.94 (95% CI: 2.80, 3.09); *p* < 0.001
	500 Hz	−0.00 (95% CI: −0.23, 0.23); *p* = 0.994	−0.00 (95% CI: −0.20, 0.20); *p* = 0.998	0.00 (95% CI: −0.20, 0.20); *p* = 0.988	0.00 (95% CI: −0.20, 0.20); *p* = 0.996
	250 Hz	−0.00 (95% CI: −0.23, 0.23); *p* = 0.996	0.00 (95% CI: −0.20, 0.20); *p* > 0.999	0.00 (95% CI: −0.20, 0.20); *p* = 0.985	0.00 (95% CI: −0.20, 0.20); *p* = 0.972
	200 Hz	0.01 (95% CI: −0.22, 0.24); *p* = 0.936	0.00 (95% CI: −0.20, 0.20); *p* = 0.979	0.00 (95% CI: −0.19, 0.20); *p* = 0.962	0.01 (95% CI: −0.20, 0.21); *p* = 0.956
	125 Hz	0.01 (95% CI: −0.21, 0.24); *p* = 0.898	0.01 (95% CI: −0.19, 0.21); *p* = 0.927	0.01 (95% CI: −0.19, 0.20); *p* = 0.948	0.02 (95% CI: −0.18, 0.22); *p* = 0.874
	100 Hz	0.02 (95% CI: −0.21, 0.25); *p* = 0.850	0.01 (95% CI: −0.19, 0.21); *p* = 0.933	0.02 (95% CI: −0.18, 0.22); *p* = 0.860	0.00 (95% CI: −0.20, 0.21); *p* = 0.964
	50 Hz	0.16 (95% CI: −0.07, 0.39); *p* = 0.172	0.08 (95% CI: −0.12, 0.29); *p* = 0.415	0.06 (95% CI: −0.14, 0.26); *p* = 0.554	0.06 (95% CI: −0.14, 0.27); *p* = 0.535
	40 Hz	**0.41 (95% CI: 0.18, 0.64); *p* < 0.001**	0.10 (95% CI: −0.11, 0.30); *p* = 0.350	0.09 (95% CI: −0.11, 0.28); *p* = 0.398	0.07 (95% CI: −0.13, 0.27); *p* = 0.490
	30 Hz	**1.08 (95% CI: 0.85, 1.31); *p* < 0.001**	**0.50 (95% CI: 0.29, 0.70); *p* < 0.001**	**0.94 (95% CI: 0.74, 1.14); *p* < 0.001**	**0.88 (95% CI: 0.68, 1.08); *p* < 0.001**
	25 Hz	**0.90 (95% CI: 0.70, 1.10); *p* < 0.001**	**0.86 (95% CI: 0.66, 1.06); *p* < 0.001**	**0.76 (95% CI: 0.53, 0.99); *p* < 0.001**	**0.90 (95% CI: 0.70, 1.10); *p* < 0.001**
	20 Hz	**0.86 (95% CI: 0.66, 1.06); *p* < 0.001**	**0.71 (95% CI: 0.52, 0.91); *p* < 0.001**	**0.82 (95% CI: 0.62, 1.02); *p* < 0.001**	**0.86 (95% CI: 0.66, 1.06); *p* < 0.001**
LF/HF (percent)	Intercept	1.39 (95% CI: 1.19, 1.58); *p* < 0.001	1.46 (95% CI: 1.27, 1.65); *p* < 0.001	1.65 (95% CI: 1.44, 1.86); *p* < 0.001	1.39 (95% CI: 1.19, 1.58); *p* < 0.001
	500 Hz	−0.00 (95% CI: −0.28, 0.28); *p* = 0.994	−0.00 (95% CI: −0.27, 0.26); *p* = 0.988	0.00 (95% CI: −0.29, 0.29); *p* = 0.996	−0.00 (95% CI: −0.28, 0.28); *p* = 0.994
	250 Hz	−0.01 (95% CI: −0.28, 0.27); *p* = 0.969	−0.00 (95% CI: −0.27, 0.26); *p* = 0.987	0.00 (95% CI: −0.29, 0.29); *p* = 0.994	−0.01 (95% CI: −0.28, 0.27); *p* = 0.969
	200 Hz	−0.01 (95% CI: −0.28, 0.27); *p* = 0.954	−0.01 (95% CI: −0.27, 0.26); *p* = 0.964	−0.01 (95% CI: −0.30, 0.28); *p* = 0.934	−0.01 (95% CI: −0.28, 0.27); *p* = 0.954
	125 Hz	−0.02 (95% CI: −0.30, 0.26); *p* = 0.881	−0.01 (95% CI: −0.27, 0.26); *p* = 0.951	−0.02 (95% CI: −0.31, 0.27); *p* = 0.899	−0.02 (95% CI: −0.30, 0.26); *p* = 0.881
	100 Hz	−0.00 (95% CI: −0.28, 0.27); *p* = 0.975	−0.02 (95% CI: −0.29, 0.24); *p* = 0.862	−0.03 (95% CI: −0.32, 0.27); *p* = 0.855	−0.00 (95% CI: −0.28, 0.27); *p* = 0.975
	50 Hz	−0.08 (95% CI: −0.36, 0.20); *p* = 0.559	−0.08 (95% CI: −0.35, 0.19); *p* = 0.563	−0.21 (95% CI: −0.50, 0.09); *p* = 0.170	−0.08 (95% CI: −0.36, 0.20); *p* = 0.559
	40 Hz	−0.09 (95% CI: −0.37, 0.19); *p* = 0.521	−0.11 (95% CI: −0.38, 0.16); *p* = 0.420	**−0.54 (95% CI: −0.83, −0.25); *p* < 0.001**	−0.09 (95% CI: −0.37, 0.19); *p* = 0.521
	30 Hz	**−1.30 (95% CI: −1.58, −1.02); *p* < 0.001**	**−1.40 (95% CI: −1.67, −1.13); *p* < 0.001**	**−1.57 (95% CI: −1.86, −1.27); *p* < 0.001**	**−1.30 (95% CI: −1.58, −1.02); *p* < 0.001**
	25 Hz	**−1.30 (95% CI: −1.58, −1.03); *p* < 0.001**	**−1.23 (95% CI: −1.49, −0.96); *p* < 0.001**	**−1.02 (95% CI: −1.31, −0.73); *p* < 0.001**	**−1.30 (95% CI: −1.58, −1.03); *p* < 0.001**
	20 Hz	**−1.24 (95% CI: −1.52, −0.96); *p* < 0.001**	**−0.97 (95% CI: −1.24, −0.71); *p* < 0.001**	**−1.25 (95% CI: −1.55, −0.94); *p* < 0.001**	**−1.24 (95% CI: −1.52, −0.96); *p* < 0.001**

Downsampled values that are different from the 1000 Hz reference-standard are bolded. Electrocardiography (ECG), blood pressure (BP), middle cerebral artery (MCA), posterior cerebral artery (PCA), standard deviation of N-N intervals (SDNN), root mean square of successive differences between heartbeats (RMSSD), low frequency (LF), and high frequency (HF).

## Data Availability

Data are available upon reasonable request to the corresponding author (J.S.B.).

## Supplementary Materials

The following supporting information can be downloaded at: https://www.mdpi.com/article/10.3390/s24072048/s1, Figure S1: Log-transformed Bland–Altman plot with 95% limits of agreement depicting the agreement of heart rate values at various downsampled frequencies compared to the “reference-standard” 1000 Hz in 54 individuals (29 females and 25 males). The mean difference is shown with a red dashed line and the 95% limits of agreement are shown with blue lines. Heart rate variability metrics were obtained from an electrocardiography (ECG) and pulse rate variability metrics from three devices using photoplethysmography (PPG) measuring blood pressure (BP) at the finger and velocity within the middle cerebral artery (MCA) and posterior cerebral artery (PCA); Figure S2: Log-transformed Bland–Altman plot with 95% limits of agreement depicting the agreement of the standard deviation of N-N intervals values at various downsampled frequencies compared to the “reference-standard” 1000 Hz in 54 individuals (29 females and 25 males). The mean difference is shown with a red dashed line and the 95% limits of agreement are shown with blue lines. Heart rate variability metrics were obtained from an electrocardiography (ECG) and pulse rate variability metrics from three devices using photoplethysmography (PPG) measuring blood pressure (BP) at the finger and velocity within the middle cerebral artery (MCA) and posterior cerebral artery (PCA); Figure S3: Log-transformed Bland–Altman plot with 95% limits of agreement depicting the agreement of the root mean square of successive differences between heartbeats values at various downsampled frequencies compared to the “reference-standard” 1000 Hz in 54 individuals (29 females and 25 males). The mean difference is shown with a red dashed line and the 95% limits of agreement are shown with blue lines. Heart rate variability metrics were obtained from an electrocardiography (ECG) and pulse rate variability metrics from three devices using photoplethysmography (PPG) measuring blood pressure (BP) at the finger and velocity within the middle cerebral artery (MCA) and posterior cerebral artery (PCA); Figure S4: Log-transformed Bland–Altman plot with 95% limits of agreement depicting the agreement of relative low frequency values at various downsampled frequencies compared to the “reference-standard” 1000 Hz in 54 individuals (29 females and 25 males). The mean difference is shown with a red dashed line and the 95% limits of agreement are shown with blue lines. Heart rate variability metrics were obtained from an electrocardiography (ECG) and pulse rate variability metrics from three devices using photoplethysmography (PPG) measuring blood pressure (BP) at the finger and velocity within the middle cerebral artery (MCA) and posterior cerebral artery (PCA); Figure S5: Log-transformed Bland–Altman plot with 95% limits of agreement depicting the agreement of relative high frequency values at various downsampled frequencies compared to the “reference-standard” 1000 Hz in 54 individuals (29 females and 25 males). The mean difference is shown with a red dashed line and the 95% limits of agreement are shown with blue lines. Heart rate variability metrics were obtained from an electrocardiography (ECG) and pulse rate variability metrics from three devices using photoplethysmography (PPG) measuring blood pressure (BP) at the finger and velocity within the middle cerebral artery (MCA) and posterior cerebral artery (PCA); Figure S6: Log-transformed Bland–Altman plot with 95% limits of agreement depicting the agreement of the low frequency to high frequency ratio at various downsampled frequencies compared to the “reference-standard” 1000 Hz in 54 individuals (29 females and 25 males). The mean difference is shown with a red dashed line and the 95% limits of agreement are shown with blue lines. Heart rate variability metrics were obtained from an electrocardiography (ECG) and pulse rate variability metrics from three devices using photoplethysmography (PPG) measuring blood pressure (BP) at the finger and velocity within the middle cerebral artery (MCA) and posterior cerebral artery (PCA); Figure S7: Representative data from one participant showing clipped pulsatile waveform data collected at 1000 Hz and subsequently downsampled to various frequencies; Figure S8: Representative data from one participant showing pulsatile waveform data captured without the true systolic peak at 1000 Hz and subsequently downsampled to various frequencies.
