# Supplementary material for: Heart Rate Variability and Pulse Rate Variability: Do Anatomical Location and Sampling Rate Matter?
Source: Sensors (Basel). 2024 Mar 23;24(7):2048. doi: 10.3390/s24072048 (PMC11013825; doi:10.3390/s24072048)
Supplement: Supplementary file 1 [file sensors-24-02048-s001.zip › Supplemental Figure Captions.pdf]

**Figure S1**: Log-transformed Bland-Altman plot with 95% limits of agreement depicting the agreement of heart rate values at various downsampled frequencies compared to the "*reference-standard*" 1000 Hz in 54 individuals (29 females and 25 males). Heart rate variability metrics were obtained from an electrocardiography (ECG) and pulse rate variability metrics from three devices using photoplethysmography (PPG) measuring blood pressure (BP) at the finger and velocity within the middle cerebral artery (MCA) and posterior cerebral artery (PCA).

**Figure S2**: Log-transformed Bland-Altman plot with 95% limits of agreement depicting the agreement of the standard deviation of N-N intervals values at various downsampled frequencies compared to the "*reference-standard*" 1000 Hz in 54 individuals (29 females and 25 males). Heart rate variability metrics were obtained from an electrocardiography (ECG) and pulse rate variability metrics from three devices using photoplethysmography (PPG) measuring blood pressure (BP) at the finger and velocity within the middle cerebral artery (MCA) and posterior cerebral artery (PCA).

**Figure S3**: Log-transformed Bland-Altman plot with 95% limits of agreement depicting the agreement of the root mean square of successive differences between heartbeats values at various downsampled frequencies compared to the "*reference-standard*" 1000 Hz in 54 individuals (29 females and 25 males). Heart rate variability metrics were obtained from an electrocardiography (ECG) and pulse rate variability metrics from three devices using photoplethysmography (PPG) measuring blood pressure (BP) at the finger and velocity within the middle cerebral artery (MCA) and posterior cerebral artery (PCA).

**Figure S4**: Log-transformed Bland-Altman plot with 95% limits of agreement depicting the agreement of relative low frequency values at various downsampled frequencies compared to the "*reference-standard*" 1000 Hz in 54 individuals (29 females and 25 males). Heart rate variability metrics were obtained from an electrocardiography (ECG) and pulse rate variability metrics from three devices using photoplethysmography (PPG) measuring blood pressure (BP) at the finger and velocity within the middle cerebral artery (MCA) and posterior cerebral artery (PCA).

**Figure S5**: Log-transformed Bland-Altman plot with 95% limits of agreement depicting the agreement of relative high frequency values at various downsampled frequencies compared to the "*reference-standard*" 1000 Hz in 54 individuals (29 females and 25 males). Heart rate variability metrics were obtained from an electrocardiography (ECG) and pulse rate variability metrics from three devices using photoplethysmography (PPG) measuring blood pressure (BP) at the finger and velocity within the middle cerebral artery (MCA) and posterior cerebral artery (PCA).

**Figure S6**: Log-transformed Bland-Altman plot with 95% limits of agreement depicting the agreement of the low frequency to high frequency ratio at various downsampled frequencies compared to the "*reference-standard*" 1000 Hz in 54 individuals (29 females and 25 males). Heart rate variability metrics were obtained from an electrocardiography (ECG) and pulse rate variability metrics from three devices using photoplethysmography (PPG) measuring blood pressure (BP) at the finger and velocity within the middle cerebral artery (MCA) and posterior cerebral artery (PCA).

**Figure S7**: Representative data from one participant showing clipped pulsatile waveform data collected at 1000 Hz and subsequently downsampled to various frequencies.

**Figure S8**: Representative data from one participant showing pulsatile waveform data captured without the true systolic peak at 1000 Hz and subsequently downsampled to various frequencies.
