# Supplementary figures and images for: Heart Rate Variability and Pulse Rate Variability: Do Anatomical Location and Sampling Rate Matter?
Source: Sensors (Basel). 2024 Mar 23;24(7):2048. doi: 10.3390/s24072048 (PMC11013825; doi:10.3390/s24072048)

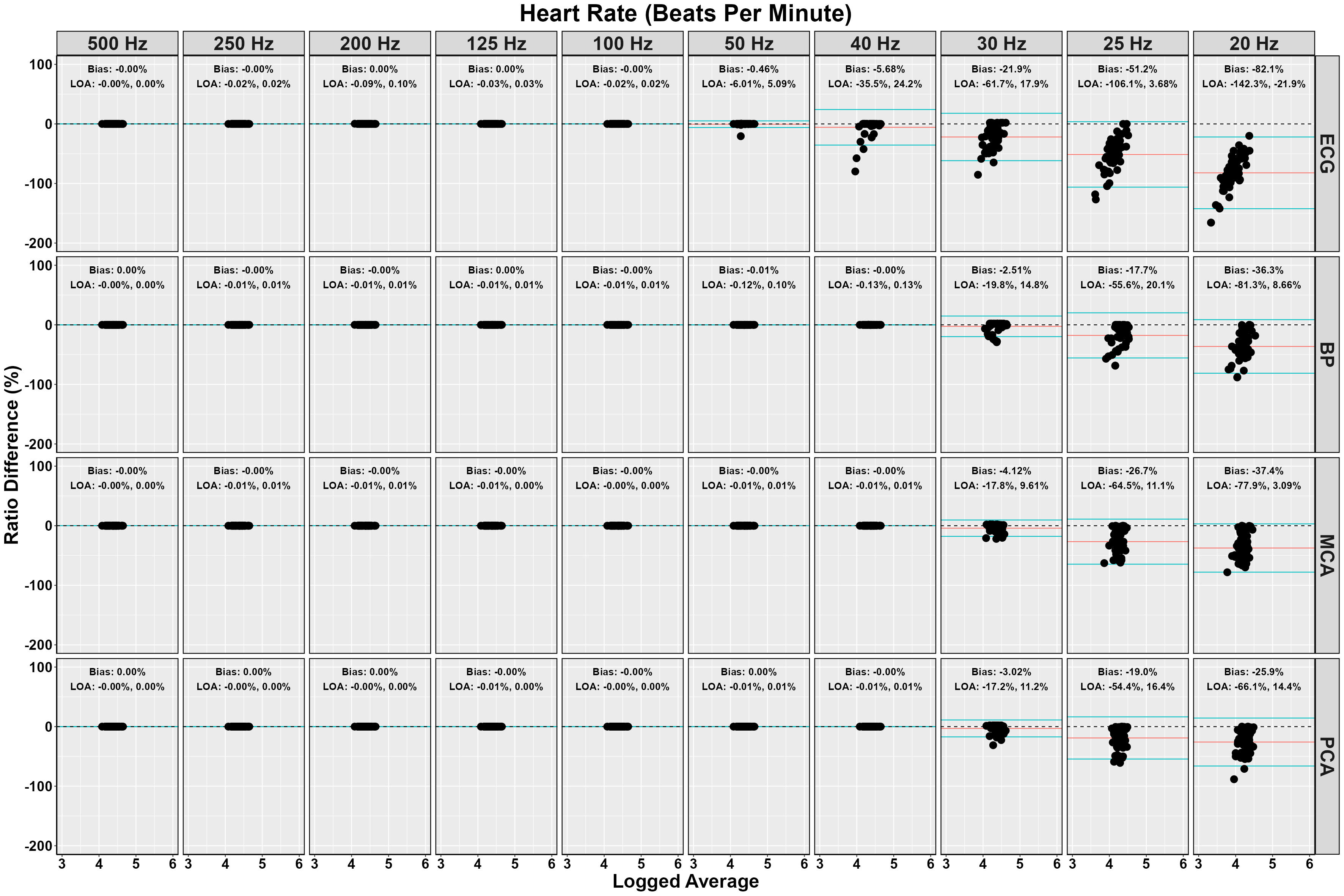

Supplement: Supplementary file 1 [file sensors-24-02048-s001.zip › Figure_S1.jpeg]

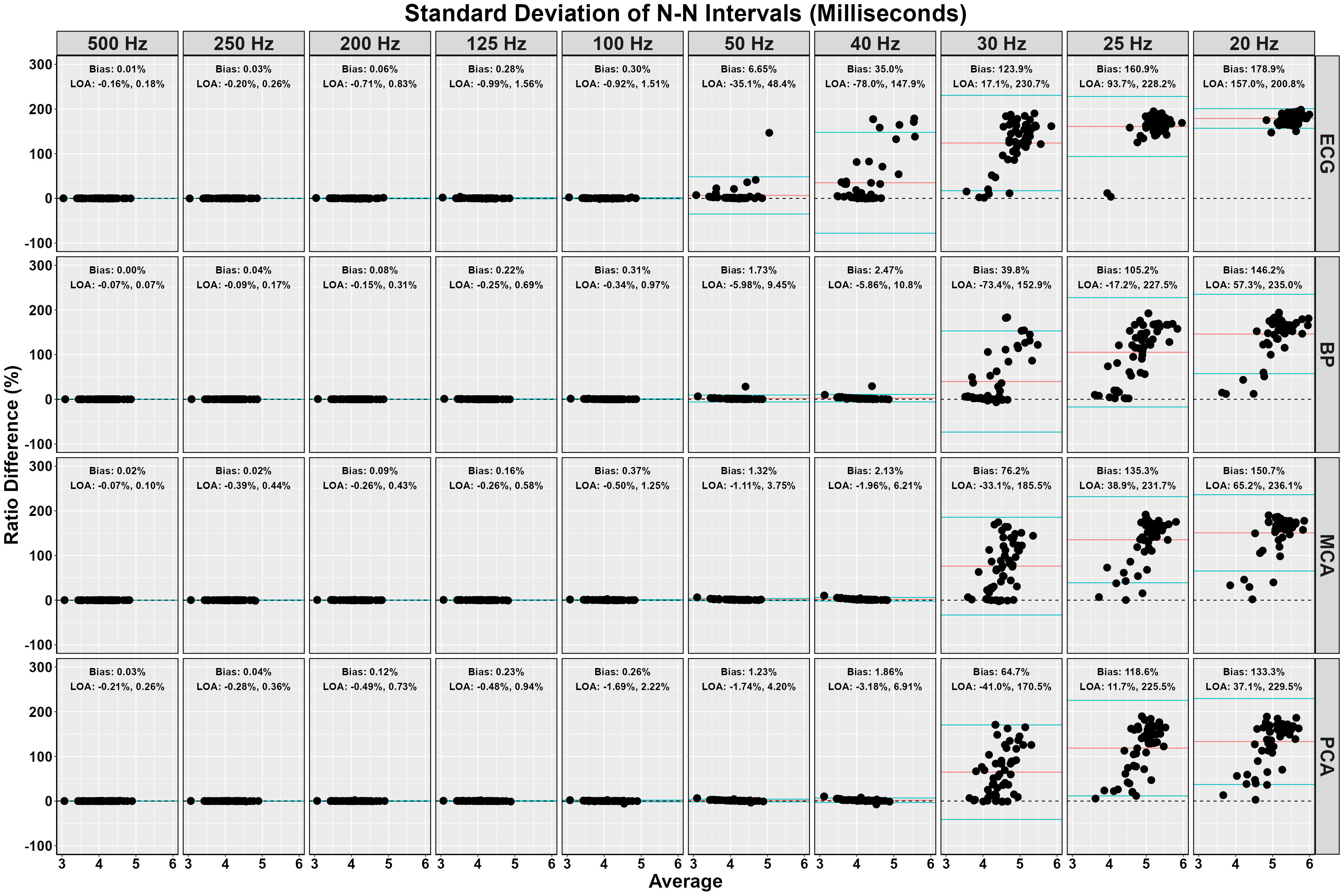

Supplement: Supplementary file 1 [file sensors-24-02048-s001.zip › Figure_S2.jpeg]

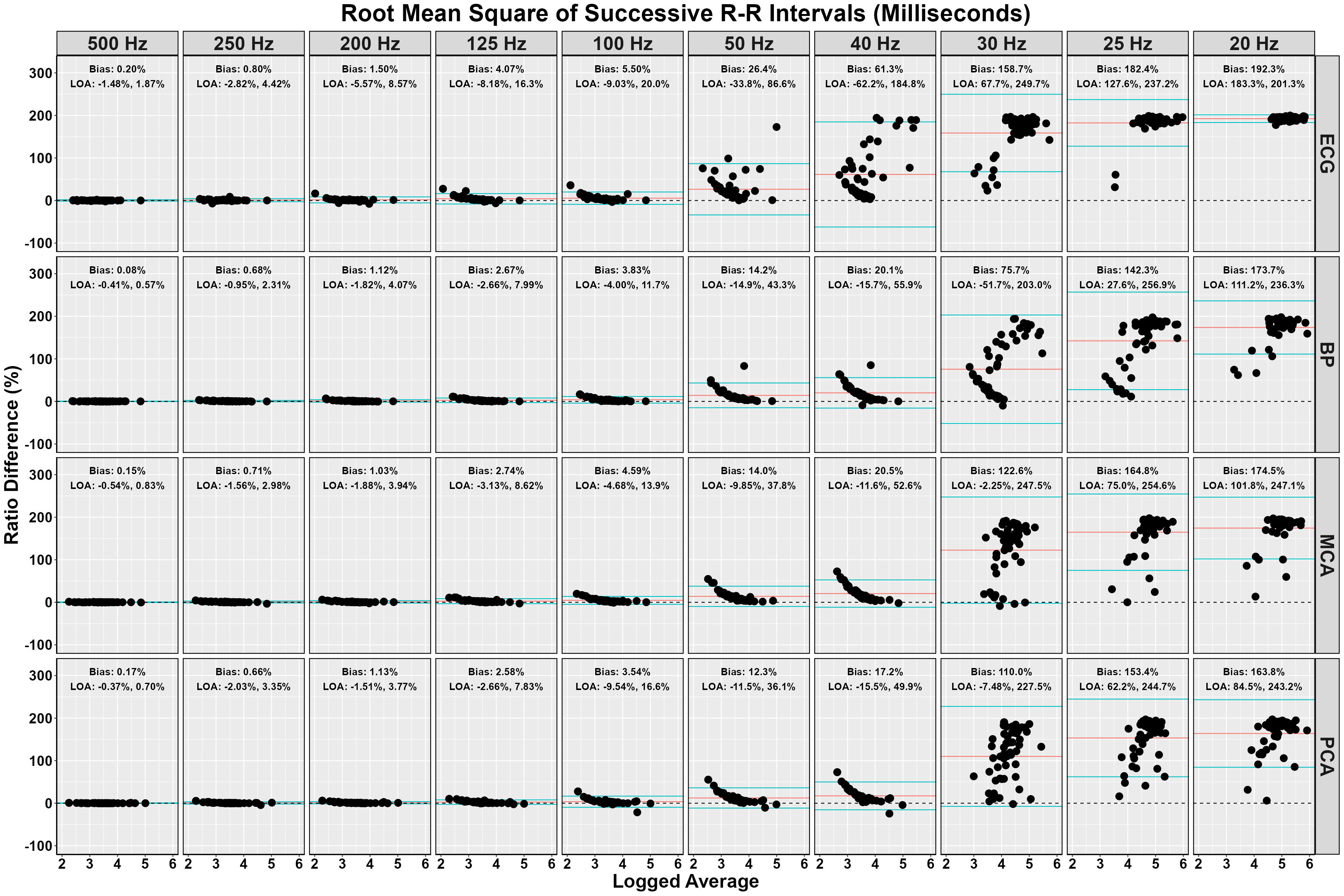

Supplement: Supplementary file 1 [file sensors-24-02048-s001.zip › Figure_S3.jpeg]

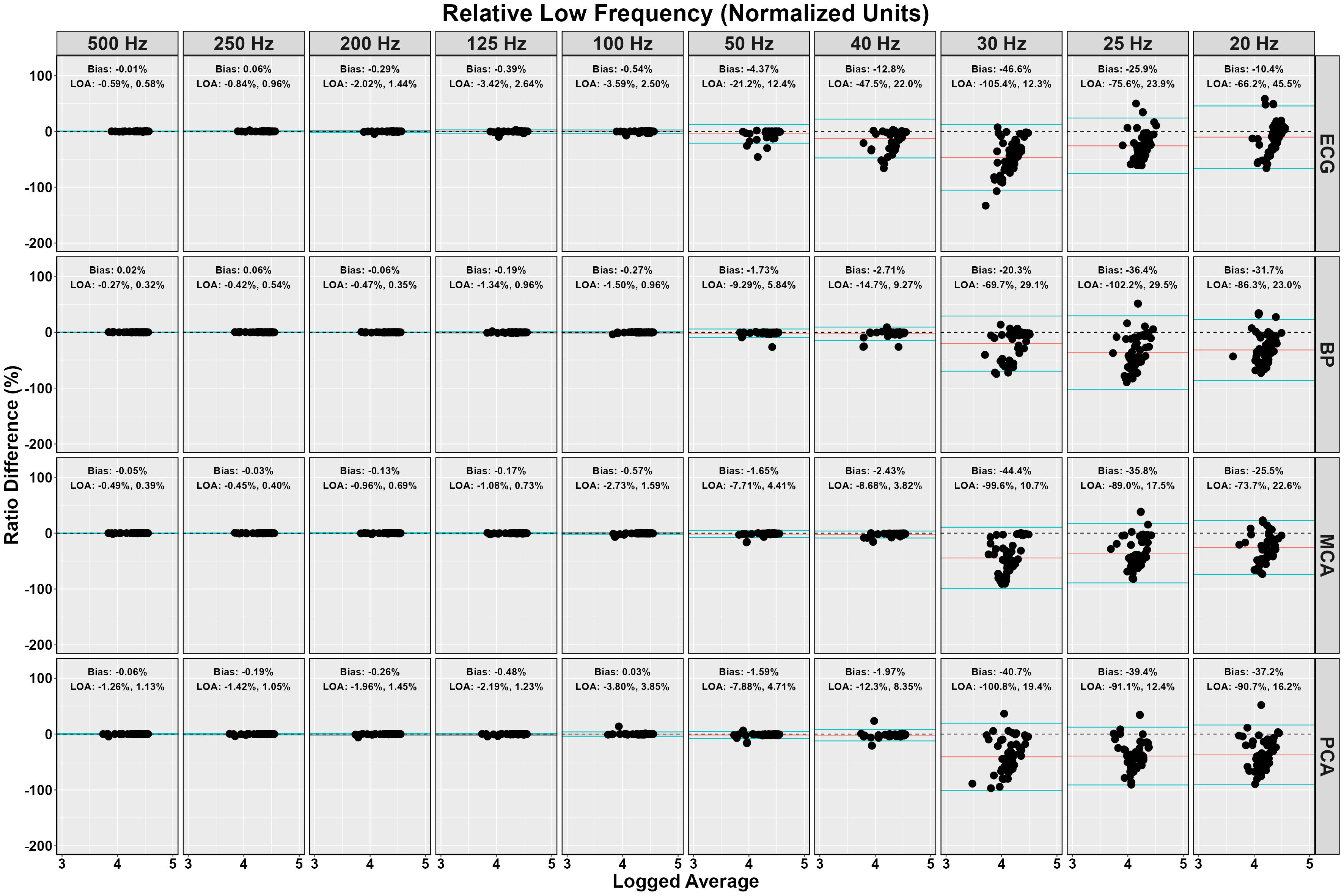

Supplement: Supplementary file 1 [file sensors-24-02048-s001.zip › Figure_S4.jpeg]

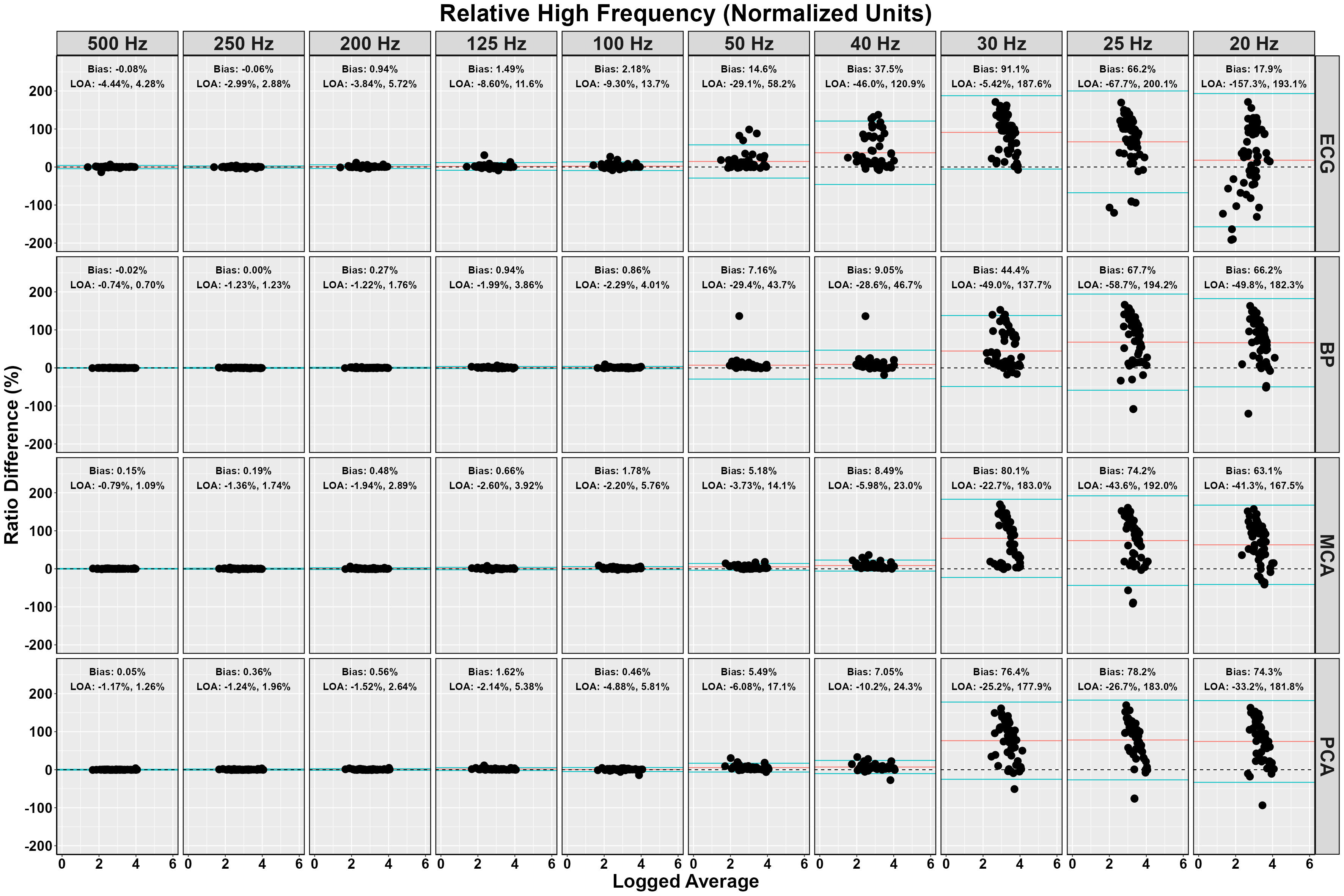

Supplement: Supplementary file 1 [file sensors-24-02048-s001.zip › Figure_S5.jpeg]

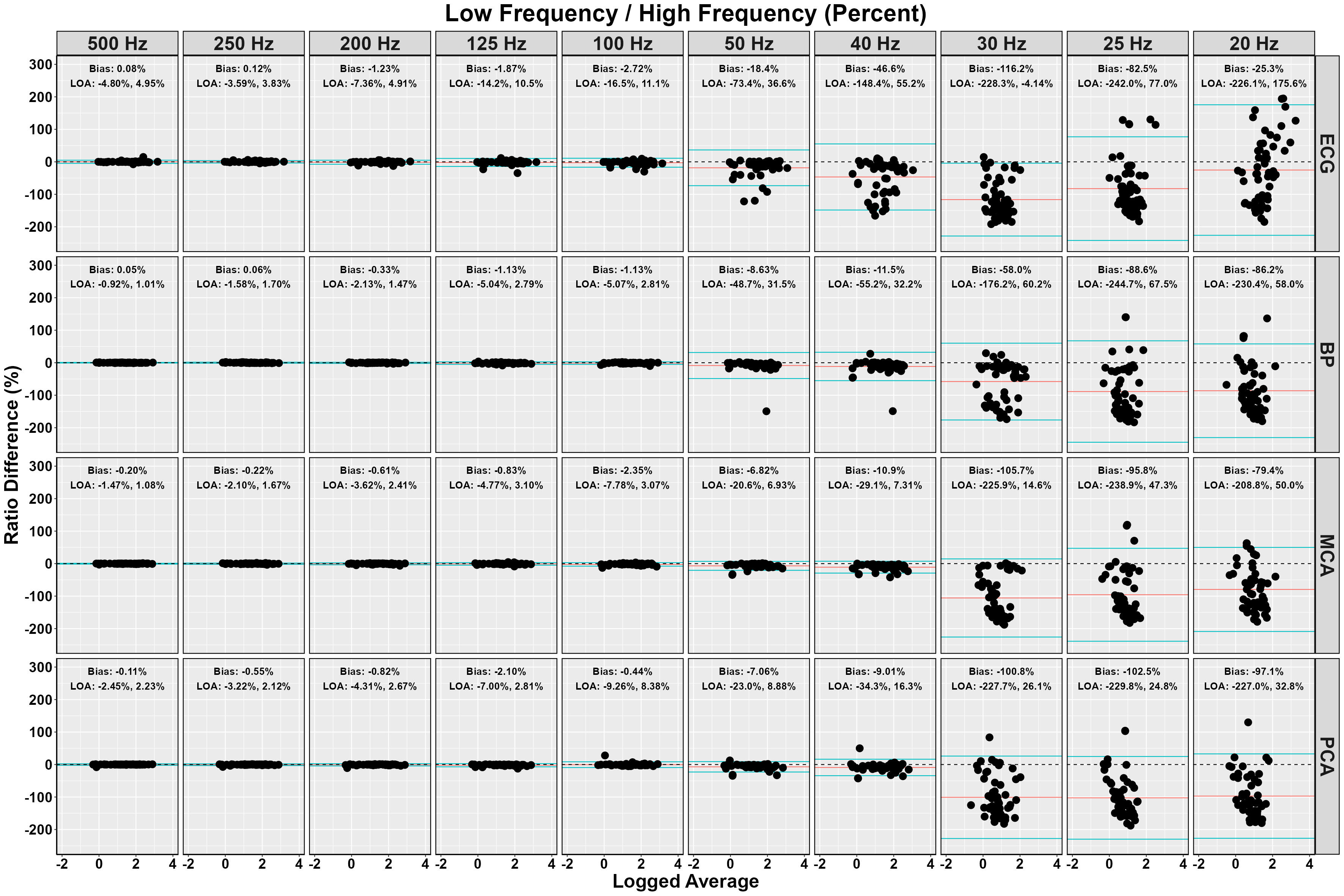

Supplement: Supplementary file 1 [file sensors-24-02048-s001.zip › Figure_S6.jpeg]

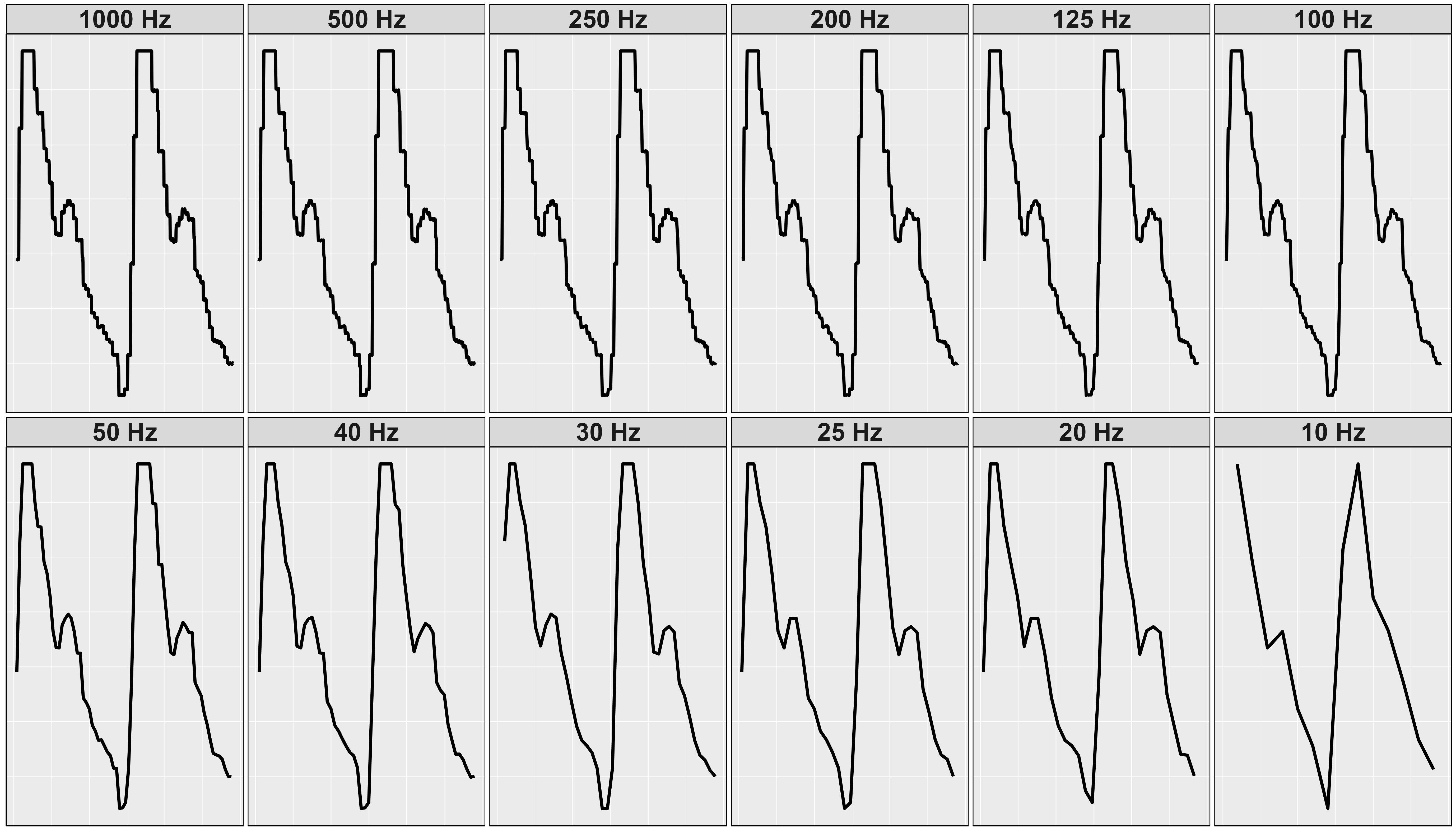

Supplement: Supplementary file 1 [file sensors-24-02048-s001.zip › Figure_S7.jpeg]
